# Self-Styled ZnO Nanostructures Promotes the Cancer Cell Damage and Supresses the Epithelial Phenotype of Glioblastoma

**DOI:** 10.1038/srep19950

**Published:** 2016-01-28

**Authors:** Rizwan Wahab, Neha Kaushik, Farheen Khan, Nagendra Kumar Kaushik, Eun Ha Choi, Javed Musarrat, Abdulaziz A. Al-Khedhairy

**Affiliations:** 1 Zoology department, College of Science, King Saud University, Riyadh 11451, Saudi Arabia; 2 Al-Jeraisy, Chair for DNA Research, Department of Zoology, College of Science, King Saud University, Riyadh 11451, Saudi Arabia; 3 Plasma Bioscience Research Center, Kwangwoon University, Seoul 139701, South Korea; 4 Department of Chemistry, Aligarh Muslim University, Aligarh U.P. India; 5 Dept. of Ag. Microbiology, AMU, Aligarh, India; 6 Baba Gulam Shah Badshah University, Rajouri, J&K, India

## Abstract

Extensive researches have been done on the applications of zinc oxide nanoparticles (ZnO-NPs) for the biological purposes. However, the role and toxicity mechanisms of ZnO nanostructures (ZnO-NSts) such as nanoplates (NPls), nanorods (NRs), nanosheets (NSs), nanoflowers (NFs) on cancer cells are not largely known. Present study was focused to investigate the possible mechanisms of apoptosis induced by self-designed ZnO-NSts, prepared at fix pH via solution process and exposed against human T98G gliomas including various cancers and non-malignant embryonic kidney HEK293, MRC5 fibroblast cells. NSts were used for the induction of cell death in malignant human T98G gliomas including various cancers and compared with the non-malignant cells. Notably, NRs were found to induce higher cytotoxicity, inhibitory effects on cancer and normal cells in a dose dependent manner. We also showed that NRs induced cancer cell death through oxidative stress and caspase-dependent pathways. Furthermore, quantitative and qualitative analysis of ZnO-NSts have also been confirmed by statistical analytical parameters such as precision, accuracy, linearity, limits of detection and limit of quantitation. These self-styled NSts could provide new perception in the research of targeted cancer nanotechnology and have potentiality to improve new therapeutic outcomes with poor diagnosis.

Over the past decade the use of inorganic metal oxides (MOs) semiconductor based nano- materials has gained interest very rapidly in the area of electronic, industries and biomedical field^1^,^2^,^3^,^4^. These materials have special attention due to their very small size, high surface area, and inexpensive as compared to the organic materials^5^. Among various semiconductor materials, the MOs of ZnO nanostructures (NSts), which exhibit wider range of NSts such as nanoplates (NPls), nanorods (NRs), nanosheets (NSs), nanoflowers (NFs) etc has special place with large applications in various optoelectronics areas for instance photooxidation, photocatalysis, solar cells, light emitting (LED), sunscreen, piezoelectric. These materials are also applied for sensors, cosmetic products, clothing, paints, and various biological systems^6^,^7^,^8^,^9^,^10^,^11^,^12^. These material has easy preparation process, which itself makes prominent, cost effective and gives various types of NSts^6^. The ZnO-NSts can be prepared via various processes such as thermal, aqueous and non-aqueous processes as described in previous reports^10^,^13^,^14^. Currently ZnO-NSts have been focused for various biological applications due to their biocompatible nature^6^. In the area of biological applications, there are enough quantity of research have been published towards the application of NPs and their role to control cancer cells growth but mechanism of cytotoxicity caused through ZnO-NSts has remained obscure^15^,^16^,^17^. Accumulating evidences suggested the reasons of cytotoxicity of ZnO-NSts through reactive oxygen species (ROS) and genotoxicity in cancer cells^15^. A recent report showed that the toxicity of cancer cells happens due to release of Zn^2+^ ions in zinc oxide solution^18^. Sharma *et al.* reported that the nanoscale zinc oxide induced DNA damage through lipid peroxidation and oxidative stress in human epidermal cells^19^. Among various types of cancers, brain, lung and human thyroid carcinomas cancer are commonly affected and considered as one of the main reason for cancer deaths. The symptoms of lung cancer are caused in the patients by primary tumor (metastasis) formation in the form of cough, chest pain, haemoptysis, dyspnea and recurrent pneumonia or bronchitis^20^,^21^. Towards this area, it has been shown that lung cancer can be successfully reduced via the utility of nanostructured materials due to the role of nanoparticles as a drug delivery carrier which reduces the nonspecific toxicity of potent anticancer drugs^22^. Higher tumor malignancy could be frequently deteriorated after cancer treatment procedures. To overcome these problems, several therapies, such as chemotherapy, radiotherapy, immune therapy, etc have been implemented to protect the cancer but the success rate of therapeutic outcomes is still not up to date^3^,^4^.

The present work demonstrates the effects of ZnO-NSts such as NPls, NRs, NSs and NFs structures against various human cancer cells. The determination of nanostructures interaction with cancer cells were also demonstrated by illustration and statistical analytical parameters. These nanostructures were used as a target material for inducing cell death (apoptosis) in malignant cells. Here, we uncover that these NSts have the potential to induce genetic damage for cancer cell death. Interestingly, these NSts significantly reduced the motility of aggressive cancer cells. These findings proposed that targeted NSts could improve existing therapeutic approaches in field of cancer nanotechnology.

## Results

### Crystalline and morphological studies of ZnO-NSts synthesized at optimized pH

**Figure 1:**
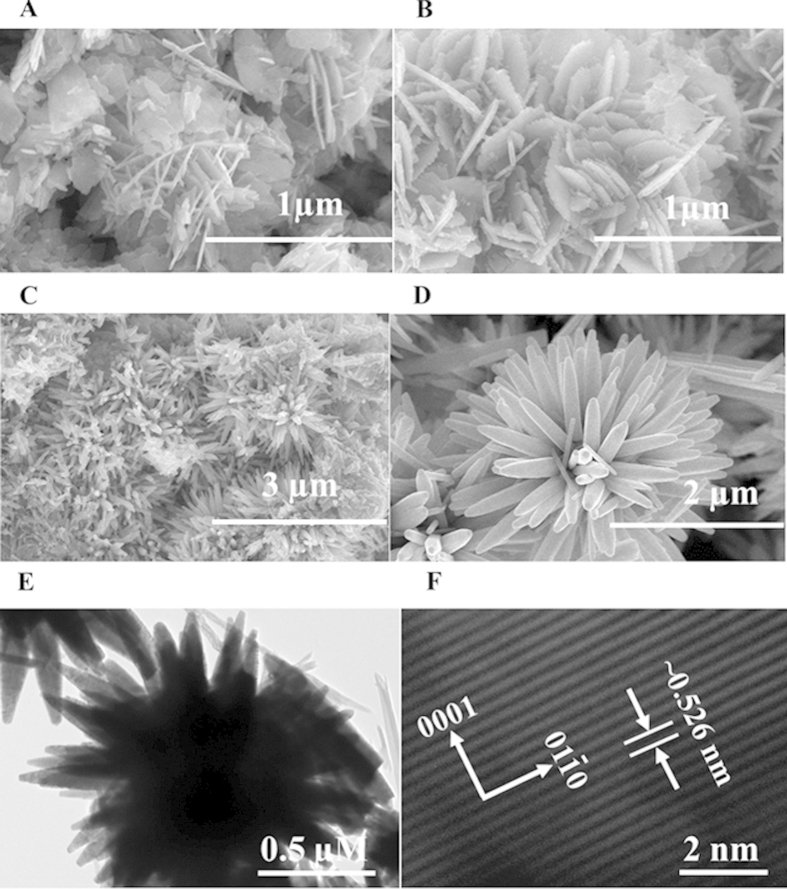
FESEM images of ZnO nanostructures. **(A)** nanoplates, **(B)** nanosheets, **(C)** nanorods, **(D)** nanoflowers, and **(E)** shows the low magnification TEM image (**E**) of ZnO nanoflowers composed with rods whereas, **(F)** presents the HR-TEM image and it displays the lattice difference between two fringes is ~0.526 nm.

The general morphology of the NSts (NPls, NSs, NRs and NFs) was analyzed using FE-SEM prepared at desired pH conditions with the use of precursor solution of zinc acetate dihydrate and sodium hydroxide. The aqueous solution of zinc acetate dihydrate and sodium hydroxide have been calibrated and optimized at pH (6, 9, 10, and 12) with the incorporation of H^+^ and OH^−^ ions (HCl & NaOH). Figure 1A,B shows the FESEM images of NPls and NSs composed with tiny NRs structure optimized at pH 6 and 7. A visible change has been observed in the morphology of ZnO-NSts with the addition alkali (NaOH) solution. As the solution gets basic OH^−^ ions from sodium hydroxide, the sheet changes to NRs (Fig. 1C) and further changed to primary flower shaped-like structure. At pH 12, the structure gets more stable and shows a complete flower shaped morphology (Fig. 1D). The diameter of each NR is ~150–200 nm whereas length is about 2–4 μm long, sharp tipped with hexagonal surface.

These NSts were further investigated by TEM microscopy at 200 kV. Figure 1E shows the low magnification TEM image of ZnO-NFs. The observations from TEM are closely related with the FE-SEM observation and clearly consistent. Figure 1B shows that the size of each NR is exactly identical dimension (~200 nm long and full array was about 2–4 μm) obtained from FE-SEM. Figure 1F shows the HR-TEM (high resolution transmission electron microscopy) image of ZnO-NFs composed with hexagonal nanorods. From the HR-TEM image, we can easily observe that the distance of lattice fringes between two adjacent planes are ~0.526 nm, which is almost identical to the lattice constant of wurtzite ZnO. The observed lattice distance from HR-TEM image, again indicates that the obtained NR of flower shaped morphology exhibit wurtzite hexagonal phase and are preferentially grown along the c-axis [0001] as shown in Fig. 1F.

### Analytical determinations of ZnO-NSts

**Figure 2:**
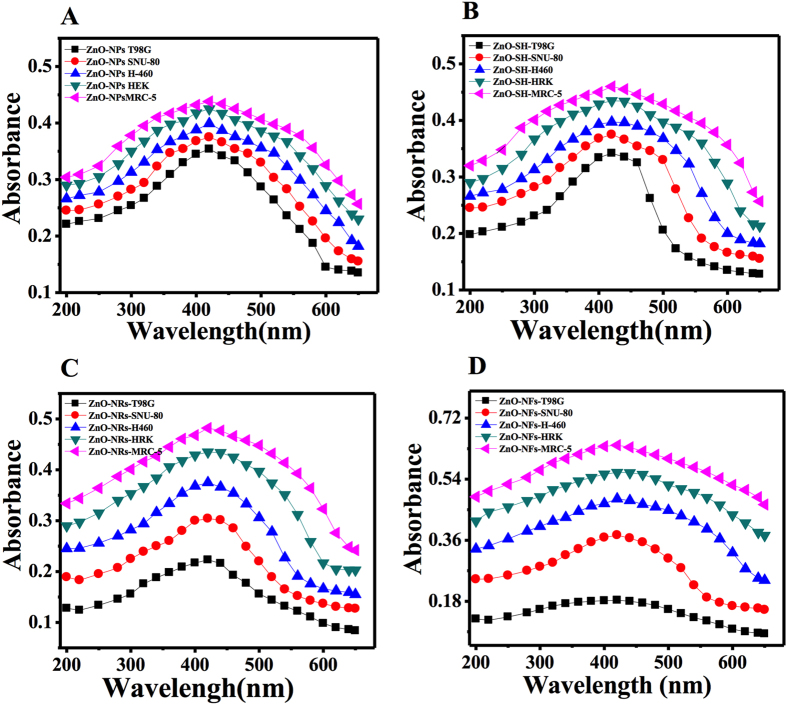
The absorption spectra of different shaped ZnO nanostructures. nanoplates **(A)**, nanosheets **(B)**, nano rods **(C)** and nanoflowers **(D)** with cancer and normal cells (T98G, SNU-80, H460, HEK and MRC-5).

The analytical performance was measured for the synthesized NPls, NSs, NRs and NFs formed at fixed pH, which are directly depends on the range of concentration of nanostructures, calculated by mathematical or statistical analytical tools as seen in Fig. 2. In the suspension solution of NSts with cells, statistical approach was applied for the appropriate choice and evaluated the capability of pH mediated NSts of ZnO and their fitness for the purpose of inhibition of growth of cancer cells. The analytical determination was studied with different shaped ZnO-NSts.

**Figure 3:**
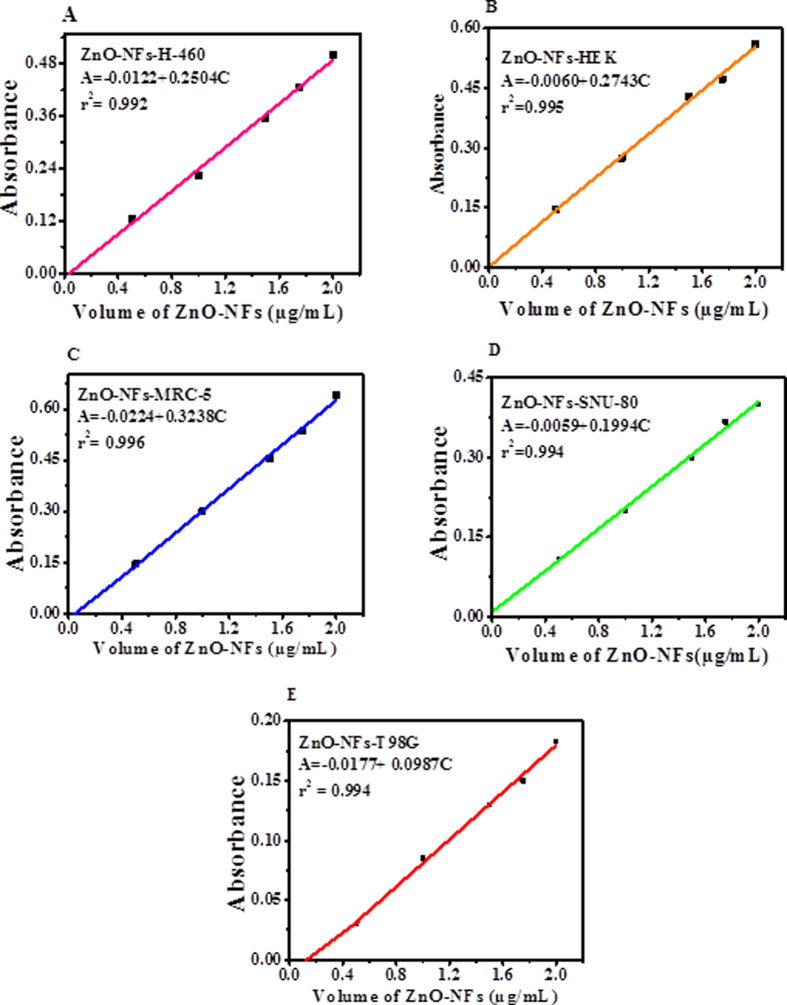
Linear calibration graph of ZnO nanoflowers with cancer and normal cells. **(A)** ZnO-NFs with H-460, **(B)** ZnO-NFs with HEK293, **(C)** ZnO-NFs with MRC-5, **(D)** ZnO-NFs with SNU-80, **(E)** ZnO-NFs with T98G.

The purposed method was based on UV-visible ZnO-NSts (NPls, NSs, NRs and NFs) spectrophotometer, which is a best tool and gives quantitative and qualitative result established by valid statistical analytical techniques under the guideline of ICH. The absorption spectra were recorded for the different shaped ZnO-NSts such as NPls, NSs, NRs and NFs etc. At initial pH 6 (grown plates), at neutral pH 7 (sheet-like structure), pH 10 (rod-like structure) and pH 12 (flower like structure) at 600 nm wavelength and the graph can be seen in Fig. 2A–D. The calibration graph was made for the ZnO-NSts (NPls, NSs, NRs and NFs) at optimized pH by plotting absorbance against concentrations (0.5–2.2 μg/mL), which gives linearity as can be seen in Fig. 3A–E. The molar absorptivity (є) of the ZnO-NSts (ZnO-NFs-T98G = 0.915 ×10^2^, ZnO-NFs-SNU-80 = 2.0 ×10^2^, ZnO-NFs-H460 = 2.50 ×10^2^, ZnO-NFs-HEK = 2.70 ×10^2 ^and ZnO-NFs-MRC-5 = 2.70 ×10^2 ^L mol^-1 ^cm^-1^ of the resulting colored sample solution indicates high sensitivity of ZnO-NSts towards cancerous cells (Table S1, supporting information). The detection limit (LOD) and quantitation limit (LOQ) was determined for known concentration of sample solution at minimum level, their empirical formula provided more reliably values to ensure that they are fit for the proposed method and confirmed this method is accurate, precise, reproducible and gives adequate results. The relative standard deviation (RSD), standard analytical error (SAE), Confidence limit at 95% confidence level (n=5) results are summarized in Table S2, supporting information. It is evident from these observations that the relative standard deviation (RSD %) in the ranges from (0.32–1.69%) is accurate and satisfactory^23^,^24^,^25^.

### Optimization and validation of the proposed method

The optimization and validation of suspension solution or analytes (ZnO-NSts) was recorded against in terms of many factual variables factors such as concentration, volume, pH, time and temperature etc, which is a common way for the analytical method that gives standardization of sample. The validation methods and their parameters clearly define the rule and regularities for the development of analytes, quality control, and each individual required the analytical data of analytes. The concentration of used ZnO-NSts (NPls, NSs, NRs and NFs) at optimized pH, are applied for to control the growth of cancer cells under optimized and validated experimental condition. The analytical procedure (within a given range from 0.5–2.2 μg/mL) has ability to obtained test results, which are directly proportional to the concentration of analyte in sample solutions. Beer’s law was obeyed for the obtained concentration range from 0.5–2.2 μg/mL therefore, the fixed concentration range for plates to flower like structure as can be seen in Fig. 3A–E. The regression analysis of calibration data obtained with the help of absorbance verses concentration, which gives the value of correlation coefficient (ZnO-NFs-T98G = 0.994, ZnO-NFs-SNU-80 = 0.994, ZnO-NFs-H460 = 0.992, ZnO-NFs-HEK293 = 0.995 and ZnO-NFs- MRC-5 = 0.996 at pH 12) with apparent molar absorptivity (є) (ZnO-NFs-T98G = 0.915 ×10^2^, ZnO-NFs-SNU-80 = 2.0 ×10^2^, ZnO-NFs-H460 = 2.50 × 10^2^, ZnO-NFs-HEK293 = 2.70 ×10^2^ and ZnO-NFs-MRC5 = 3.20×10^2 ^L/mol/ cm respectively. The detection limits (LOD) and quantitation limit (LOQ) were found to be 0.172: 0.523 for ZnO-NFs-T98G, 0.176:0.535 for ZnO-NFs-SNU-80, 0.213:0.645 for ZnO-NFs-H460, 0.170:0.517 for ZnO-NFs-HEK293 and 0.146:0.443 for ZnO-NFs-MRC-5 μg/mL respectively. To evaluate intra-day and inter-day precisions, analysis for ZnO-NFs at three concentration levels (0.5, 1.5 and 2.0 μg/mL) were carried out within the same day and five consecutive days. The intra-day and inter-day RSD values ranged from pH 12 ZnO-NFs-T98G for (0.60–1.12% and 0.89–1.46%), ZnO-NFs-SNU-80 (0.32–0.43% and 0.81–1.13%), ZnO-NFs-H-460 (0.30–0.63% and 0.049:0.90%), ZnO-NFs-HEK293 (0.71–1.05% and 0.72–1.69%), ZnO-NFs-MRC-5 (0.60–0.95% and 1.20–1.18%) respectively (Table S2, supporting information). The RSD (%) obtained were quantitative for pH 12 indicating the good accuracy of the proposed method analyzed by spectrophotometric method^23^,^24^,^25^.

### Inhibitory effects of ZnO-NSts on cancer and normal cells

In this study, we have compared four different types of ZnO-NSts (ZnO-Pls, ZnO-NSs, ZnO-NRs, ZnO-NFs) on three malignant and two non-malignant cell lines. To check the effectiveness of ZnO-NSts, we have treated both normal and cancer cells by different concentrations of NSts and their cytotoxicity was assessed using MTT proliferation assay. The MTT cell proliferation assay has been widely accepted as a reliable way to measure the cell proliferation rate and conversely when metabolic events lead to apoptosis or necrosis. Table S3 (supporting information) shows the IC_50_ values of NSts on the T98G brain cancer, H460 lung cancer, SNU-80 thyroid cancer, non-malignant HEK kidney and MRC-5 lung fibroblast cell lines. The data obtained by MTT assay showed that all NSts have inhibitory effects on the growth of cancer cells in a dose-dependent manner. All NSts effectively inhibited the growth of T98G, H460, SNU-80, HEK293 and MRC-5 cells, with their IC_50_ values ranging from 38.26 to 185.93 μg/mL. All NSts were found to inhibit 50% T98G glioma cell growth in the range from 36.80–48.62 μg/mL after 24 hr treatment. Interestingly, our data indicated that all NSts were found to be less cytotoxic to non-malignant normal HEK293 and MRC5 cells at moderate concentrations (187.5–11.71 μg/mL) as shown in Table S3, supporting information. IC_50_ values of the NSts for HEK293 and MRC-5 cells were in the range from 86.62–185.15μg/mL. As we mentioned earlier, that MTT assay shows that T98G cells were severely affected by the NSts treatment as dose-dependent manner. These experiments indicate that inhibition of T98G cancer cell growth by NSts may be due to either growth arrest or cell death. Collectively, these results clearly demonstrate that all NSts are effective to block cancer cell growth but more prominent effect was seen by ZnO-NRs exposure on cancer cells rather than on non-malignant cells, which may be by increasing the mitotic or interphase death.

**Figure 4:**
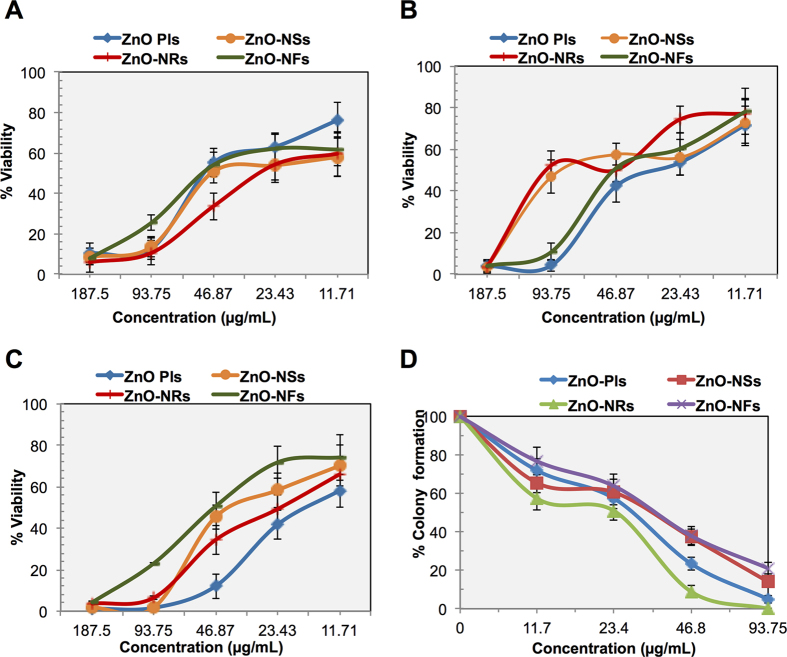
Inhibitory effects of nanostructures on cancer cells. **(A–C)** Growth kinetics of T98G cells at 24, 48 and 72 h after treatment by ZnO-NSts (NPls, NSs, NRs and NFs) at 11.71–187.5 μg/mL concentrations. Untreated cells are taken as control and all values given as mean (±SD) of three independent experiments, n = 3. **(D)** Effect of ZnO nanostructures (ZnO-NPls, ZnO-NSs, ZnO-NRs, ZnO-NFs) on colony forming capacity of exponentially growing T98G cell lines studied by clonogenic assay at 11.71–93.75 μg/mL concentrations. Data presented are mean values from three independent observations, n = 3. Error bars represent the ± SD (n = 3). Student’s *t*-test was performed, *p < 0.05, ^δ^*p* < 0.01, and ^#^*p* < 0.001.

Figure 4A–C shows the growth kinetics of the T98G cells. Data obtained by growth kinetics assay showed that the all NSts have an inhibitory effect on T98G cells that is dependent on exposure time. The cells were treated at 11.71 to 187.50 μg/mL concentrations of nanostructures. Cell suspensions were made in media layers of 4 mm thickness in 6 well plates. As a note, the cells exposed to ZnO-NSs treatments show less effect than those exposed to a ZnO-NRs treatment. The maximum effect was seen after ZnO-NRs exposure, by inhibiting the cell growth from 42–96% at 72 h at all 5 concentrations. Whereas in case of ZnO-Pls exposure on T98G cells, we found that 29.87–98.49% cells were died and their viability was 1.51–70.13%, at 72 hr respectively after treatment. ZnO-Pls showing incubation time dependent effect on cells, maximum effect is obtained only at after 48 h however showed no such effect before 48 hr of incubation. However ZnO-NFs is also showing incubation time dependent effect on the growth of T98G cells and having maximum inhibitory effect only at 48 hr of incubation rather than 24 or 72 h after incubation. Overall, all nanostructures inhibits the growth of T98G cells in a dose and incubation time dependent manner. These experiments indicate that NSts inhibit T98G cancer cell growth which may be due to either growth arrest or mitotic cell death.

**Figure 5:**
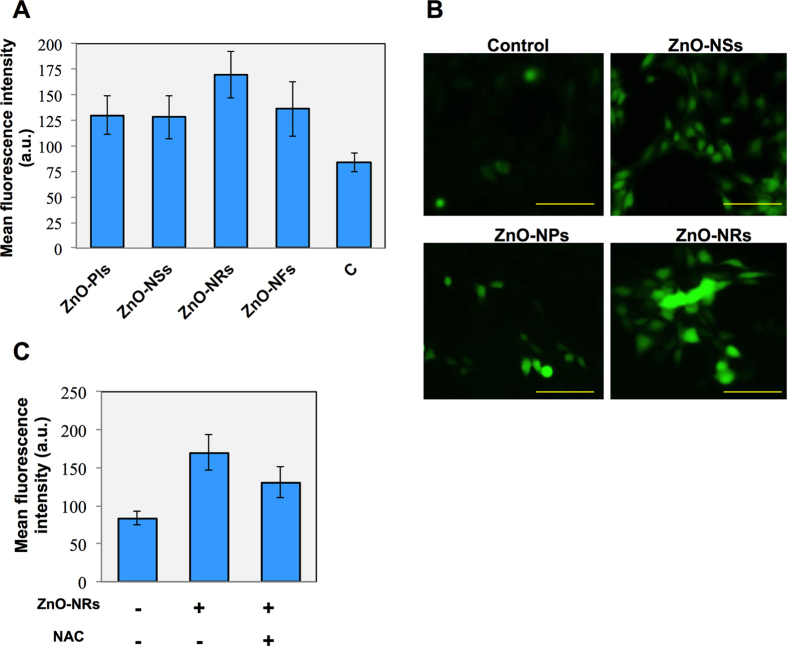
Nanostructures accumulate ROS inside cells is essential factor for cell death. (**A**) Effects of ZnO-NSts (NPls, NSs, NRs, NFs) on the reactive oxygen species (ROS) level in T98G cell lines. ROS values (in MFI, mean florescence intensity) presented are mean ± SD (n = 3). (**B**) Representative images of ZnO-NRs treated T98G cells after staining with DCFHDA dye. Each figure has scale bar of 50 μm. (**C**) ROS level in T98G cells after ZnO-NRs treated T98G cells in the presence of NAC (10 μM, N-acetyl L-cysteine, well known ROS inhibitor). Error bars represent the ± SD (n = 3). Student’s *t*-test was performed, *p < 0.05, ^δ^*p* < 0.01, and ^#^*p* < 0.001.

Figure 4D shows the effect of NSts on the colony forming capacity of T98G brain cancer cells. To this end, we performed a clonogenic assay to confirm the colony formation inhibition results due to NSts treatment. Only a fraction of seeded cells retains the capacity to produce colonies after cytotoxic compound treatment. Clonogenic assay or colony formation assay is an *in vitro* assay based on the ability of a single cell to grow into a colony. This *in vitro* clono- genicity method was recognized as a valid surrogate assay for tumor growth *in vivo*. As NSts concentration increases, the treatment enhances cell death and inhibits the colony formation capability of the T98G cell population. Our data shows that all NSts are capable of inhibiting the colony formation and survival of exponentially growing T98G cells in concentration-dependent manner, after treatments with different concentrations (11.71–93.75 μg/mL) as evidence by the reduction in the number of colonies formed (Fig. 4D). It was observed in the Fig. 5A–C, that the survival fraction of T98G cells has been drastically decreased after NSts treatment. Even at low dose, 11.71 μg/mL exposures of ZnO-Pls, ZnO-NSs, ZnO-NRs and ZnO-NFs shows significant decline in colony survival and their % colony survival was found to be 71.77, 65.32, 57.25 and 76.61 respectively. However, the significant drastic decline in their colony survival percentage could be observed after exposure to 23.43 μg/mL concentration of nanostructures. ZnO-Pls and ZnO-NRs show higher colony formation inhibitory effect on T98G cells, at all doses. Remarkably, ZnO-NRs showed maximum inhibition at doses 11.7, 23.4, 46.8 and 93.7 μg/mL exposures with percentage colony survival 57.25, 50.80, 8.87 and 0 respectively. Collectively, these results indicate that these NSts treatments are efficiently inhibiting the clonogenicity of glioma cells at all the effective concentrations.

### Nanostructure accumulated intracellular ROS is essential factor for cell death

Next we sought to check the factors which are responsible for NSts induced cell growth inhibition and observed cell death. For this purpose, first we checked the ROS formation inside the cells. Figure 5 shows ROS measurement in NSts treated T98G brain cancer cells. The decreased level of MTT formazan with growth inhibition and increased level of clonogenic inhibition might lead to increased oxidative stress that would compromise the survival fraction leading to cell death in T98G cells. Here, we found that all NSts enhanced ROS production at 24 hr inside the T98G cells. Treatment by NPls, NSs, NRs and NFs increases the DCF fluorescence by 47.38% (MFI-130), 45.96% (MFI-128), 87.38% (MFI-170) and 53.64% (MFI-136) respectively at 1.46 μg/mL concentration (Fig. 5A,B), when compared to untreated control (MFI-83.6).Notably, maximum effect was seen by ZnO-NRs in T98G cells. To further confirm this effect, we treat the T98G cells by NRs in the presence of well-known ROS inhibitor, NAC (10 μM, N-acetyl L-cysteine, Sigma Aldrich). NAC is a antioxidant and prevents cell apoptosis or death induced by reactive species. NAC is added 4 hour before nanostructure exposure to cell culture. As expected, addition of NAC significantly (*p*< 0.05) blocked the ROS generation in ZnO-NRs treated T98G cells which clearly indicate that ROS accumulation is necessary for NSts induced cell death effect (Fig. 5C).

### Caspases plays a crucial role in nanostructures induced apoptotic cancer cell death

**Figure 6:**
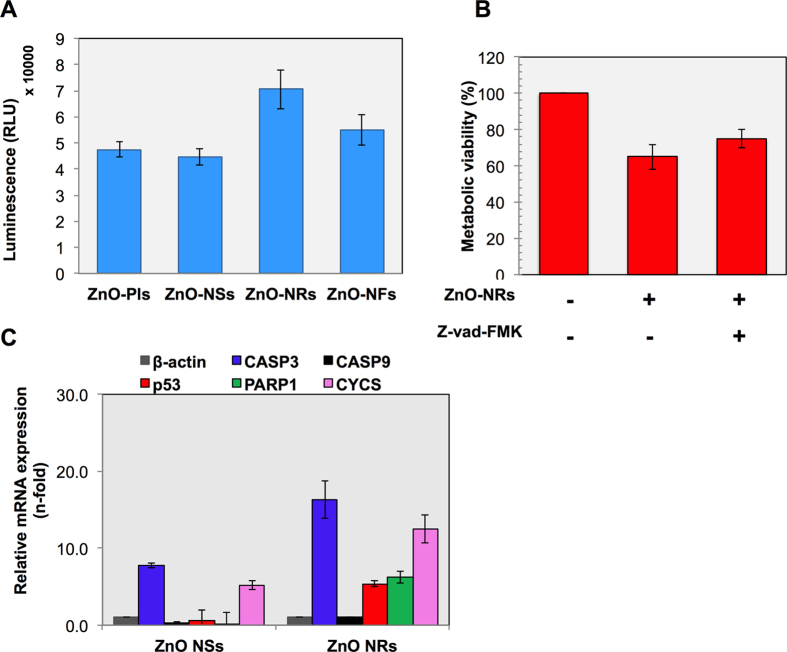
Caspases plays a crucial role in nanostructures induced apoptotic cancer cell death. (**A**) ZnO-NSts (NPls, NSs, NRs, NFs) induced activation of caspase 3/7 in T98G cell line. Apoptosis was assessed by measuring active caspase-3/7 using the Promega Caspase-3/7 assay kit and relative luminescence units were abbreviated as RLU in y-axis. (**B**) Cell viability was assessed using MTT assay in presence of pan-caspase inhibitor (100 μM, Z-vad FMK) after 24 hr followed with ZnO NRs exposure. (**C**) mRNA expression of various apoptosis related genes were analyzed using real time PCR. Experiments were performed in triplicate. Error bars represent the ± SD (n = 3). Student’s *t*-test was performed, *p < 0.05, ^δ^*p* < 0.01, and ^#^*p* < 0.001.

Next, to assess the effect of NSts on the activity of Caspase-3/7 in brain cancer cells, we treated the T98G cancer cells with all NSts at a dose of 1.46 μg/mL. It is broadly accepted that the apoptotic cell death of mammalian cells is closely associated with activation of executioner caspases which has important roles as early biochemical markers for apoptosis. To detect the caspases, we used Caspase-Glo® 3/7 assay kit (Promega) that uses a luminogenic substrate containing the DEVD sequence which is selective for caspase-3 and -7. All detection steps were performed using as manufacturer’s protocol. Following 24 hr NSts treatment, the caspases activity in both untreated and treated cancer cells were measured using luminometer as shown in Fig. 6A. Caspases activity level detected in untreated control cells corresponded to the portion of apoptotic cells present in the naturally growing population due to natural aging. It is worth-mentioning here that ZnO-NRs show the highest caspases activity among all the NSts treated in T98G cancer cells. To confirm further the potential involvement of caspases in observed cell death by nanostructures, we pretreated the T98G cells with pan-caspase inhibitor Z-VAD-FMK (100 μM, R & D systems, Minneapolis, MN, USA) and then treated with NRs at same concentration. Treatment with Z-VAD-FMK significantly (*p*< 0.05) attenuated the cell death induced by the ZnO-NRs treatment (Fig. 6B) in T98G cancer cells when compared to only ZnO-NRs-treated group. Therefore, the Z-VAD-FMK protected the cells from apoptosis induced by the ZnO-NRs treatment.

To extend our observations, we next investigated that NSts induced cell death is apoptotic or not, we checked apoptosis-related gene expression such as caspase 3 (CASP3), caspase 9 (CASP9), p53, poly ADP ribose polymerase (PARP), cytochrome C (CYTS) by real time PCR analysis. Notably, our results show that all above genes were enhanced after ZnO-NRs treatment. Interestingly, CASP3 (executioner caspases) was much elevated after ZnO-NRs treatment, signifying its main role in induced apoptotic cell death in cancer (Fig. 6C). These findings suggest that the NRs treatment may induce apoptosis through the caspase-dependent pathway.

**Figure 7:**
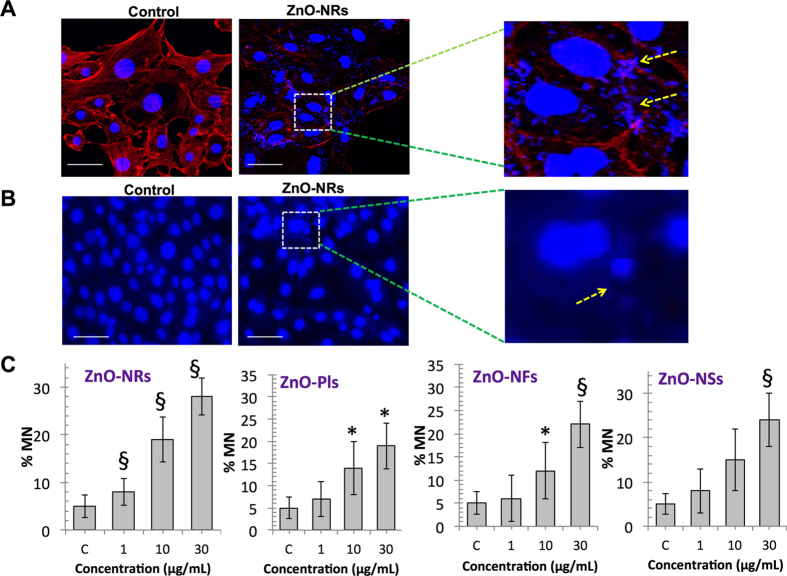
Nanostructure disrupts the cytoskeleton and genetic material in glioma cells. (**A**) Immunofluorescence assays using phalloidin rhodamine (5 units/ml, Invitrogen) were performed to visualize the cytoskeleton (F-actin); cell nucleus was counterstained with DAPI (4',6-di amidino-2-phenylindole) in ZnO-NRs treated T98G cells. Each figure has scale bar of 10 μm. (**B,C**) Micronucleus (MN) assay was performed to visualize cell micronuclei in ZnO-NRs treated T98G cells and micronucleus was counted using microscopy and representative MN frequency was shown in all nanostructures treated glioma cells. Error bars represent the ± SD (n = 3). Student’s *t*-test was performed, *p < 0.05, ^δ^*p* < 0.01, and ^#^*p* < 0.001.

When ZnO-NRs treated T98G cells morphology was examined using immunofluorescence, observed that after NRs exposure the surviving cells adhered to the surface show that actin fibers became shortened and thinner in T98G cells and nucleus become enlarged and swollen and was fragmented (Fig. 7A). The enlarged images indicated that actin fibers became shortened and localized near cell membrane. To look further closely into the nucleus, we performed micronucleus (MN) assay after all NSts treatment. Maximum micronuclei were formed after ZnO-NRs treatment (Fig. 7B). However, another NSts exposure also revealed the increased micronuclei formation in exposed T98G cells. Based on above results, we conclude here that caspases induced PARP cleavage resulting DNA damage is crucial for NSts induced apoptotic cell death.

### Nanostructures suppresses the invasiveness of glioblastoma through EMT

**Figure 8:**
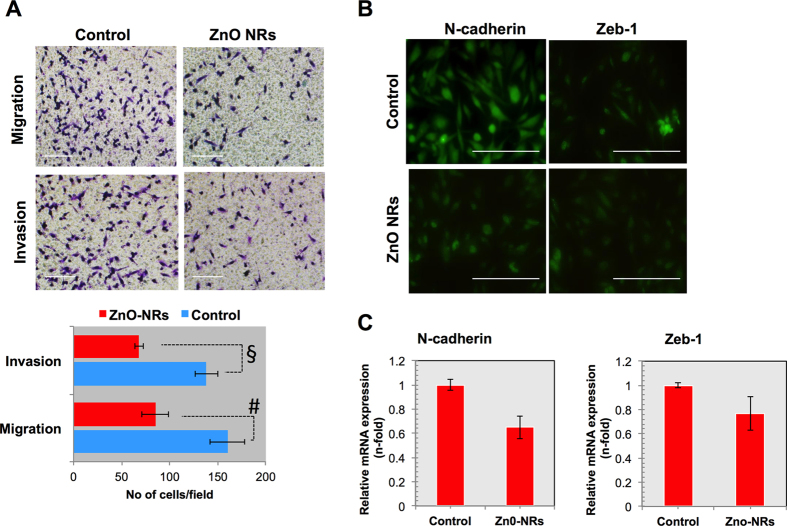
Nanostructures suppress the invasiveness of glioblastoma through decreasing EMT phenotype. (**A**) Migration and invasion assay in transwells after treatment of glioma cells that are treated with ZnO-NRs (1.46 μg/mL) in glioma cells. (**B**) Immunocytochemistry for EMT markers such as N-cadherin (1:200) and Zeb-1 (1:100) in glioma cells after ZnO-NRs treatment. Each figure has scale bar of 50 μm. (**C**) qRT PCR analysis for mRNA levels of N-cadherin and Zeb-1 in glioma cells after ZnO-NRs treatment. Experiments were performed in triplicate and repeated two times. Error bars represent the ± SD (n = 3). Student’s *t*-test was performed, *p < 0.05, ^δ^*p* < 0.01, and ^#^*p* < 0.001.

In line with agreement of above consisting results, we next determined that which mechanism is involved in NSts effect as a cancer suppressor. The epithelial-to-mesenchymal transition (EMT) is the process in which epithelial cells change their cell polarity and adhesion property and increase their invasiveness and migration capacity. Invasiveness is a characteristic feature of aggressive cancers, and this intricate property can be considered as an intense investigation for some time. An emerging concept suggests that EMT phenomenon involved in cancer progression^26^ and has been considered to regulate invasiveness of cancer cells at the tumor–stroma interface^27^. Based on these studies, we thought to gain insight in the process of EMT in glioma cells after NRs exposures and characterized the migratory and invasive properties. We observed that ZnO-NRs significantly (*p*< 0.05) decreased the migration and invasion in glioma cells (Fig. 8A). However, various molecules are involves in EMT process, we checked neuronal cadherin (N-cadherin) and transcription factor zinc finger E-box-binding homeobox1 (Zeb-1) which is mostly activated during EMT process. Staining and gene expression analysis clearly shows that these markers are significantly attenuated after ZnO-NRs treatment in glioma cells (Fig. 8B,C). Taken together these results suggest that NRs has the potential to suppress the epithelial phenotype in cancer cells to inhibit the cancer progression.

## Discussion

In the family of metal oxides, ZnO which has wide verity of nanostructure such as nanoparticles, nanorods, nanowire, nanobelts, nanonuts, nanotube, nanoring, nanobridges, nanonails, nanocubes, nano sheet, sea-urchin, nanoflowers and nanofan shaped nanostructures, and the shape evolutions strongly depend on the pH value during growth of NSts^28^,^29^,^30^,^31^,^32^,^33^,^34^,^35^. All these nano and micro- structures were prepared either by low temperature synthesis or thermal evaporation methods^36^. The nano& micro scaled nanoproducts exhibit wide applicability in various industrial and biomedical applications such as drug delivery, DNA damage, cytotoxicity, cancer etc; due to their high surface area to volume ratio, which enhances wide catalytic properties^37^. Here, we have described the utility of diverse shaped NSts such as NPls, NSs, NRs and NFs in growth inhibition of cancer cells. The obtained nanoscale structures were well characterized with FE-SEM, TEM equipped with the HR-TEM to know the general morphology of NSts and their crystallinity for the formation of nanostructures. The FE-SEM and TEM images, which showed that the prepared NSts having diverse shaped structure such as the early staged grown plates synthesized at pH = 6, whereas a small deviation have been seen as the pH was increased to neutral solution pH=7. The sheet-like structure converted to aligned plate and rod like structure. As the value of zinc oxide suspension solutions pH changes from acidic to basic (6–9), grown nanosheet structures turns to separate as well as structurally arranged-nanorods (Fig. 1), but when the pH value of the solution increased to pH =10, only rod-like structure was observed with hexagonal shape and tapered tip (Fig. 1). As the solution pH value increases to 11, NRs are seen forming a flower like structure and synthesis at pH =12, results in a complete flower like morphology composed to sharp tipped hexagonal nanorods was formed with nearly smooth surface with an average diameter of each nanrods 150–200 nm, whereas length goes about 2–4 μm long, sharp tipped with hexagonal surface in spherical shaped flowers structures. The high resolution TEM (HR-TEM) again confirms that the grown structures having lattice spacing (~0.526 nm), crystalline and equal to pure wurtzite phase ZnO. The NSts of ZnO is an optically active material, which exhibit similar band gap to the commercial zinc oxide powder (3.37eV) and a typical characteristic band of pure wurtzite ZnO^38^.

The pH value has always been an important parameter to finalize nanostructured products for better understand (effect of pH) and their growth of different morphology of ZnO-NSts^38^ and has capability to inhibit the growth of cancer and normal cells such as T98G, SNU-80, H-460 and multiplications with HEK293 and MRC-5 fibroblast cells. It is also well known that the solutions pH support to determine the quantitative, qualitative response for the prepared pH mediated NSts for instance fitness by statistical, numeric and graphical optimization tools, and facilitate their optimization of multiple responses with other parameters such as concentration, temperature and time etc. Therefore, the suspension solution of pH based nano-product appears in phase formation, particles size and morphology of nanostructures, analyzed via analytical method using UV-vis spectrophotometric technique. As evaluation of analytical method indicates excellent performance characteristics are accuracy, specificity, linearity measure, solution working range, limit of quantitation (LOQ), limit of detection (LOD), intermediate precision and reproducibility etc; validated by as per the guidelines of International Conference of Harmonisation (ICH)^24^,^25^,^39^,^40^,^41^,^42^,^43^. The target of statistical analysis provides broad knowledge of system suitability and characteristics performance, which relates to the multi-dimensional data’s operator, obtained from the process used in experimental design. Hence, this method is an adequate, accurate, precise, acceptable behavior and based on criteria of analytical study to control morphology of nanostructures. On the basis of physical morphology and characteristics, we can postulates that all the cells have small pores which helps, the NSts to enter into the inner membrane of cells. As we know that the prepared nanostructures have property to enter into cells (~20 μm) very easily due to very small size (~150–200 nm) as compared to the cancer cells (~20 μm)^44^,^45^. The high density of NSts in the liquid system strongly favors the rapid formation of agglomerates and it’s assumed that these agglomerates of NSts destroy the cells^45^.The ROS is another factor, which is produced in solution of NSts with cells and are responsible to form the free radicals in the solution and these free radicals penetrates the outer wall of the cells and enter to inner wall of the membrane. These free radicals when reacts with the organelles, enzymatic changes occurred and it leads to the disorganization of the cells and cell contents. The oxidative stress plays an important role in the toxicity with NSts and suggested that the extreme generation of ROS through NSts reduces the cellular antioxidant capacity^46^,^47^. The phenomenon why ZnO-NSts are responsible to regulate the growth or destroy the cell organelles and their biochemical and enzymatic changes of cancer cells is under investigation and it needs further study to investigate the role of ZnO-NSts in cancer cells^45^. Here these findings shows that the nanostructured (NPls NSs, NRs, and NFs) are more potent material against cancer cells, which is may be due the small size of NSts and it can be easily entered to cell membrane^48^,^49^. Several published literature employed metal oxides nanostructures applied at a high concentrations for the toxicities studies, which may be difficult for the human exposure. We believe that these newly biocompatible NSts would be helpful to control the growth cancer cells at lower concentration and have no adverse effect on the body^50^.

## Conclusion

The obtained results of NSts (NPls, NSs, NRs, and NFs) treatment against human T98G glioma, H-460 lung carcinoma and SNU-80 thyroid carcinoma cells were compared with non-malignant HEK293 and MRC-5 fibroblast cells. All these NSts were more efficacious on cancer cells and less toxic to non-malignant human HEK293 and MRC-5 cells at effective concentrations. Overall, according to the MTT, growth kinetics, clonogenic and ROS measurement assay results, we can conclude that NSts treatment has a significant inhibitory effect on glioma cell growth and also created oxidative stress in the cells which is confirmed by using ROS inhibitors NAC. Production of reactive oxygen species (ROS) is initiated after treatment with NSts, followed by apoptotic cell death, which is confirmed by Caspase-3/7 assay. Based on resulting experimental data’s, apoptosis occurred in the cells treated with NSts as the level of these executioner caspases was increased many folds over the basal level of untreated cells. This could be due to ROS in cells, which has been shown to be able to induce cell death in several human cancer cells. Decreased viability of glioma cells strongly suggests that oxidative stress induced by NSts resulted in the observed cell death or apoptosis. The importance of such work lies in the possibility that the next-generation NSts might be more efficacious in context of anticancer nano-agents. However, further investigations relating to the size, structure and activity of the NSts as well as their stability under physiological conditions such biological fluids will be helpful and may result in the design of more potent anticancer nano-agents for therapeutic use. Specially, we found the exact concentration of NSts especially NRs and their quantity quantified by statistical analytical parameters for the maximum control of cancer cells. Further studies are in due course to understand, why NSts are responsible for cancer cell death.

## Materials and Methods

### Synthesis and characterizations of pH mediated self-styled ZnO structures (ZnO-NPls, NSs, NRs and NFs)

The synthesis of ZnO-NSts were performed with the use of zinc acetate dihydrate [(Zn(CH_3_COO)_2_.2H_2_O), Sigma-Aldrich] as a precursor and sodium hydroxide (NaOH) employed as reduction material via chemical solution route. The aqueous solution of zinc acetate dihydrate [(Zn(CH_3_COO)_2._2H_2_O) was calibrated for the desired pH values with using alkali NaOH and HCl (1M) in 100 mL distilled water (DW)as previously described^38^. The general structural morphology of the obtained nanostructure was examined via field emission scanning electron microscopy (FE-SEM) & transmission electron microscopy (TEM). For FE-SEM analysis, synthesized powder was sprayed on carbon tape and coated with conducting material. The nanostructured powder was fixed on a sample holder and analyzed at room temperature. For detailed information about NSts, we performed high resolution TEM (HR-TEM) microscopy. To this end, nano structures of white nanoscale powder of ZnO was sonicated in ethanol solvent for 10–15 min and then a carbon coated copper grid (400 mesh) was dipped in a suspension solution of NSts and dried at room temperature and crystalline, structural morphology property were analyzed at 200 kV.

### Human cell culture maintenance and nanostructures treatment

Human cancer cell lines T98G (glioblastoma), H460 (lung carcinoma), SNU-80 (thyroid cancer) HEK293 (normal embryonic kidney) and MRC5 (normal lung fibroblast) were obtained from KCLB (Korean Cell Line Bank) and cultured in DMEM media (Hyclone, USA) supplemented with 10% fetal bovine serum (FBS, Gibco) and 1% penicillin/streptomycin solution (Sigma-Aldrich). All cultures were maintained at 37 °C, 95% relative humidity and 5% CO_2_. Cells were seeded in 96-well plates at a concentration of 1 × 10^4 ^cells/well in 200 μL of complete media and incubated for 24 hour (hr) at 37 °C in 5% CO_2_ atmosphere to allow for cell adhesion. Stock solutions (3 mg/mL) of NSts diluted in PBS and were filter-sterilized, and then further diluted upto 2.92 μg/mL in incomplete media for treatment against all five malignant and non-malignant cell lines. A 100 μL solution of NSts (made in cell growth media) was added to a 100 μL solution of fresh medium in wells to give final concentrations of 1500–2.92 μg/mL. Control group containing no NSts material was run in triplicates during each assay.

### Cell proliferation assays

All malignant and non-malignant cell lines, which were treated with various NSts were used for the MTT (3-(4, 5-dimethylthiazol-2-yl)-2, 5-diphenyltetrazolium bromide) assay to check cytotoxicity. After desired time points, 20 μL MTT solutions (5 mg/mL) were added to each well and plates were incubated for 3 hr. Following incubation, the medium was removed and the purple formazan precipitate in each well was sterilized in 100 μL DMSO. Absorbance was measured using Biotek Synergy HT microplate reader at 540 nm and percentage (%) viability was calculated as described previously^51^,^52^ Results are expressed as inhibitory concentration 50 (IC_50_) were obtained by fitting values to TREND dose−response curves using the routines provided by MicroSoft Excel.

For growth kinetics assay, T98G cells were seeded at 10^4^ cells in the 100 mm cell culture plate and their proliferation kinetics were studied at different incubation times (24, 48 and 72 hr) after NSts treatment. Following trypsinization, total cells were counted per plate microscopically using a trypan blue (0.4%) and hemocytometer. In addition, we have tested the colony formation ability in NSts treated T98G cells. This assay is widely accepted to determine cell reproductive death after treatment with ionizing radiation, but we can also be used to determine the effectiveness of drug molecules. For the clonogenic assay, T98G cells having NSts treatment with the indicated concentrations and plated in the 60 mm cell culture dish after 24 hr. After 8–10 days, the colonies were fixed with methanol and stained with crystal violet. Colonies were quantified using cell counting and the surviving fraction (SF) is calculated. All assays were performed in three independent sets of duplicate tests.

### Detection of intracellular ROS

ROS levels were determined by incubating the cells with 10μM dichlorodihydrofluorescein diacetate (DCFH-DA, Molecular probes, USA) for 30 min at 37 °C. After getting into the cell and cleaved by intracellular esterase, this compound resulting in the formation of fluorescent compound 2’, 7’-dichlorodihydrofluorescin (DCFH). Stained cells were washed twice in PBS, trypsinized and immediately measured fluorescence using plate reader (synergy HT, BioTek) with excitation at 485 nm and emission at 528 nm. The data was analyzed from four individual wells in three independent plates and normalized to control wells.

### Executioner caspases detection

Following NSts treatments, T98G cells were subjected to Caspase-3/7 activity measurement with Caspase-Glo 3/7 assay kit (Promega, Madison USA) after 24 hr of incubation. Briefly, 100 μL of Caspase-Glo reagent was added to each well, the content of well was gently mixed with a plate shaker at 300–500 rpm for 30 seconds (sec). The plate was then incubated at room temperature for 2 hr. The luminescence of each sample wells was measured by the plate luminometer (Synergy HT, BioTek).

### Cytogenetic damage analysis

Micronuclei were analyzed in nanostructures treated T98G cells, previously seeded onto coverslips. Following treatment, 24 hr post treatment, cells were fixed with 4% paraformaldehyde (Sigma-aldrich) and subjected to DAPI staining (5 μg/mL, Sigma-aldrich). Only bi or tri nucleated cells were recorded, which was confirmed by visualization of the cell cytoplasm with phase-contrast microscopy (Ti-U, Nikon).

### *In vitro* migration and invasion assays

Migration and invasion assays were performed by using Transwell boyden chamber (Corning) according to the manufacturer’s instructions. Pre-coated filters (8-μm pore size, 10 mg/mL growth factor-reduced matrigel) were rehydrated with 100 μL medium. Then, 2 × 10^4 ^T98G cells in 200 μL media were placed in the upper part of each chamber, whereas the lower chamber were filled with 800 μL cell culture media containing 10% FBS. All procedures were determined by a previously described protocol^53^. Cells were photographed by phase contrast microscopy (Ti-U, Nikon). For migration assay, we used the Transwell chambers with inserts that contained the same type of membrane without the matrigel coating.

### RNA extraction and real time PCR

Cells total RNA was isolated using the Trizol reagent (Invitrogen) and cDNA was synthesized using Superscript II reverse transcriptase kit (Invitrogen). To detect the apoptosis-related (Cas3, Cas9, p53, PARP and CYTS) and EMT (N-cadherin and Zeb-1) markers gene expression, significant changes in the mRNA expression were calculated by means of real-time RT-PCR. Real-time PCR was performed on a CFX96™ Real-Time System with a, BioRad machine using IQSYBR Green Supermix (BD Biosciences). For apoptosis genes, the primer assays used in this study are listed in supporting information, Table S4 (supporting information) and the levels of gene expression relative to β-actin were determined as described by RT^2^ qPCR Primer Assay Handbook. For EMT-related gene expression, we used: human *N-cadherin*, forward primer, 5′-CACTG CTCAGGACCCAGAT-3′, and reverse primer, 5′- TAAGCCGAGTGATGGTCC-3′human *Zeb-1*, forward primer, 5-GCACCTGAAGAGGACCAGAG-3′, and reverse primer, 5′- TGCATCTGGTGTTCCATTTT-3; *GAPDH*, forward primer, 5′- ATGTTCGTCATGGGTG TGA ACCA-3, and reverse primer, 5′- TGGCAGGTTTTTCTAGACGGCAG-3′) and relative expression values were calculated using the comparative Ct method as described previously^54^.

### Immunocytochemistry

Cells were fixed with 4% paraformaldehyde and permeabilized with 0.1% Triton X-100 in phosphate-buffered saline (PBS). Following fixation, cells were incubated at 4 °C overnight with mouse polyclonal anti-human N-cadherin (1:200), rabbit polyclonal anti-human anti-zeb-1 (1:200), primary antibody in PBS with 1% bovine serum albumin and 0.1% Triton X-100. Immunostained cells were visualized using Alexa Fluor 488-conjugated anti-mouse and anti-rabbit secondary antibodies (Invitrogen). For actin fibres staining, we used phalloidin dye (5 units/mL, Phallotoxins, Invitrogen) and nuclei were counterstained by Prolong antifade gold reagent (Molecular probes, USA) and visualized under a fluorescence microscope (Ti-U, Nikon).

### Statistical analysis

Data is expressed as a mean ± SD. Statistical analysis was performed by non-parametric Student *t*-tests. Results were considered significant when *p* < 0.05.

### Statistical determination of NSts suspended in malignant and non-malignant cells

For the determination and analytical validation of the ZnO nanostructures against cancer and normal cells, cell suspension were further diluted (10 times) and analyzed in terms of analytical parameters such as linearity, range, accuracy, precision and limits of detection (LOD) and limit of quantitation (LOQ) were analyzed. The nanostructures statistical performance were also estimated in terms of mean standard deviation & variance, relative standard deviation, coefficient of correlation, regression line, variance errors in the slope and intercept and confidence limit for the slope and the intercept^39^,^40^,^41^,^42^,^43^ (See supporting information for more detailed description).

## Additional Information

**How to cite this article**: Wahab, R. *et al.* Self-Styled ZnO Nanostructures Promotes the Cancer Cell Damage and Supresses the Epithelial Phenotype of Glioblastoma. *Sci. Rep.* **6**, 19950; doi: 10.1038/srep19950 (2016).

## Supplementary Material

Supplementary Information
