# Supplementary material for: Self-Styled ZnO Nanostructures Promotes the Cancer Cell Damage and Supresses the Epithelial Phenotype of Glioblastoma
Source: Sci Rep. 2016 Jan 28;6:19950. doi: 10.1038/srep19950 (PMC4730157; doi:10.1038/srep19950)
Supplement: Supplementary Information [file srep19950-s1.pdf]

Supporting Information

Self-Styled ZnO Nanostructures Promotes the Cancer Cell Damage and Supresses the Epithelial Phenotype of Glioblastoma

Rizwan Wahab[1,2†]*, Neha Kaushik[3†], Farheen Khan[4], Nagendra Kumar Kaushik[3]*

Eun Ha Choi[3], Javed Musarrat[5] and Abdulaziz A.Al-Khedhairy[1]

[1]*Zoology department, College of Science, King Saud University, Riyadh 11451, Saudi Arabia*

[2]*Al-Jeraisy, Chair for DNA Research, Department of Zoology, College of Science, King Saud University, Riyadh 11451, Saudi Arabia*

[3]*Plasma Bioscience Research Center, Kwangwoon University, Seoul 139701, South Korea*

[4]*Department of Chemistry, Aligarh Muslim University, Aligarh U.P. India*

[5]*Department of Agricultural Microbiology, Faculty of Agricultural Sciences, Aligarh Muslim University, Aligarh 202002, India.*

[†] These authors have contributed equally to this work.

***Corresponding Authors:** RW (rwahab@ksu.edu.sa) & NKK

(kaushik.nagendra@kw.ac.kr)

Statistical analytical determination of nanostructures suspended in cancerious and non cancerious cells

For the determination and analytical validation of the zinc oxide nanostructures against suspension solution of cells were further diluted and analyzed in terms of analytical parameters.

Optimization and validation of solution with analytical techniques

A method validation studies, including the prerequisites, assumptions and formulae used in the design of an experimental plan. The validation method should be applied to analytical techniques and verify the method works reliably in their hands. Typical validation parameters are described and calculate with the help of analytical studies for examples [1].

- Linearity
- Range
- Accuracy
- Precision
- Limits of detection and quantitation

Linearity:

Linearity demonstrated by the using a minimum of five standards whose concentration span 80-120% of the expected concentration range. The linearity of a method should be established by the inspection of the plot of the instrumental response versus the initial concentration of analyte and evaluated by appropriate statistical methods. The calculation of the regression line using least square method were record the important executive recognition such as the slope of regression line, intercept, correlation coefficient (r^2) and residual sum of squares.

Range:

The specified range is consequent from the linearity studies. The range of the proposed procedure is the interval between the upper and lower concentration (amount) of analyte in the sample and covered the target amount in the operational samples to be measured. The range established by analytical method has proper levels of precision, accuracy and linearity.

Accuracy:

The accuracy is defined as closeness of the test result to the true value. Accuracy analyzed known concentrations of sample solution minimum at three concentration level covering the precise range.

Precision:

According to International conference on harmonization (ICH), the precision is the closeness of agreement between a series of measurements obtained from multiple sampling of the same identical sample under the prescribed conditions and may be considered at three levels such as repeatability, intermediate precision and repro ducibility.

Limits of detection and quantitation:

The lowest concentration of analyte in sample solution can be detected and determine by limit of detection (LOD). Limit of detection “limit test” parameter not to be quantified but it is significantly different from that of a blank. On other hand limit of quantitation (LOQ) is a parameter of “determination test” and can be defined as the lowest conc-entration of the analyte that can be precise and quantified with adequate precision and accuracy. The most common definition of LOD and LOQ is the analyte concentration for which the signal exceeds that for a realistic analytical blank by three and ten times of the standard deviation, respectively.

The lowest concentration of analyte in sample solution can be detected and determine by limit of detection (LOD). Limit of detection “limit test” parameter not to be quantified but it is significantly different from that of a blank. On other hand limit of quantitation (LOQ) is a parameter of “determination test” and can be defined as the lowest concentration of the analyte that can be precise and quantified with adequate precision and accuracy. The most common definition of LOD and LOQ is the analyte concentration for which the signal exceeds that for a realistic analytical blank by three and ten times of the standard deviation, respectively.

Limit of detection $$LOD = 3.3 \times \frac{S_0}{b}$$

Limit of quantitation $$LOQ = 10 \times \frac{S_0}{b}$$

Where (S_0) = Standard deviation of the blank, (b) Residual standard deviation (RSD) of the calibration line, or standard deviation of the intercept.

The obtained calibration curve of slope (b), and standard deviation of the intercept (S_0) analyzed at the time to validate level, acceptable accuracy and precision under the quantified operational conditions of proposed method. The limit of quantitation is around of triple the limit of detection. The analytical method was applied to verify the proposed methods works reliability. Typical validation parameters are used for accurate concentration of analytes (ZnO-nanostructures) or sample solution and calculate with the help of statistical parameters as per guidelines of International Conference of Harmo nisation (ICH) [1].

Statistical analysis:

The analytical determination of ZnO nanostructures are used for the suspension cells study and estimated by statistical analysis. The performance of the method appraisal includes the following parameters [2-4]:

- Mean
- Standard deviation & variance
- Relative standard deviation
- Coefficient of correlation
- Regression line
- Variance
- Errors in the slope and the intercept
- Confidence limit for the slope and the intercept

Mean. It is the sum of all the measurements divided by the number of measurements. It is calculated by the following expression:

$$\bar{x} = \sum_i x_i / \mathrm{n}$$

Standard deviation, variance & standard analytical error. The most useful measure of spread is the standard deviation, S.D. This is defined by the formula:

$$S.D. = \sqrt{\frac{(x_i - \bar{x})^2}{(n-1)}}$$

The square of S.D. is a very important quantity known as the variance which is useful in propagation of error.

The standard analytical error (SAE) of the mean is calculated by SAE = S.D. / √n

Relative standard deviation. It is calculated by

$$RSD = {}^{SD}/_{\bar{x}} \times 100$$

The RSD (also called coefficient of variation), the units of which are percent is an example of relative error.

Coefficient of correlation. When using instrumental methods, it is necessary to carry out a calibration process by using a series of samples (standard) each having a known concentration of analyte. Linearity is judged by correlation coefficient, ‘r’, which can be

calculated for a calibration curve to ascertain the degree of correlation between the measured instrumental variable and the sample concentration.

$$r = \frac{n\Sigma x_i y_i - \Sigma x_i \Sigma y_i}{\sqrt{[n\Sigma x_i^2 - (\Sigma x_i)^2][n\Sigma y_i^2 - (\Sigma y_i)^2]}}$$

Where n = number of data points

As a general rule, $0.90 < r < 0.95$ indicates a fair curve, $0.95 < r < 0.99$ as a good curve, and $r > 0.99$ includes excellent linearity.

Regression line. The best straight line through a series of experimental points is that line for which the sum of the squares of the deviation of the points from the line is minimum. Besides, determining a straight line, uncertainties in the use of calibration graph for analysis of unknown samples can be specified by this method of least squares. The equation of the straight line is

$$A = a + b\,C$$

Where A = instrumental response (i.e. absorbance), b = slope, a = intercept, C = concentration of the standards. To obtain the regression line A on C, the slope 'b' of the line and the intercept 'a' on the y-axis are given by the following equations:

$$b = \frac{\Sigma x_i y_i - [(\Sigma x_i \Sigma y_i)/n]}{\Sigma x_i^{\,2} - [(\Sigma x_i)^2/n]}$$

$$a = \bar{y} - b\bar{x}$$

Where $\bar{x}$ = mean of all the values of x_i and $\bar{y}$ = mean of all the values of y_i.

Errors in the slope and the intercept. The determination of errors in the slope (b) and intercept (a) of the regression line may be calculated by first calculating S_0 (standard deviation of the calibration line) from the following equation:

$$S_o = \sqrt{\Sigma(y_i - \bar{y})^2/(n-2)}$$

where $\bar{y}$ values are obtained from calculated regression line for given values of x; once the value S_o has been obtained, both the standard deviations of the slope S_b and the intercept S_a can be obtained from the following equations

$$S_b = S_o\sqrt{\Sigma(x_i - \bar{x})^2}$$

$$S_a = S_o\sqrt{\Sigma x_i^{\,2}/n\Sigma(x_i - \bar{x})^2}$$

Confidence limit for the slope and the intercept. It determines whether the slope and/or intercept of a line differ significantly from a particular or predicted value. It can be calculated [1] in the following manner:

- $b \pm t\, S_b$ (for slope)
- $a \pm t\, S_a$ (for intercept)

where t = tabulated 't' value at desired confidence level for (n-2) degrees of freedom.

References

1. International Conference on Harmonisation, Harmonized Tripartity Guideline, Validation of Analytical Procedures, Text and Methodology, Q2 (R1), November (2005).
2. Karnes H.T., Shiu G and Shahi V. P., Validation of bioanalytical methods, Pharm Res 8(4), 421-426 (1991).
3. Limentani G.B., Ringo M.C., Ye F., Bergquist M. L and McSorley E.O., Beyond the t- test: statistical equivalence testing, Anal Chem 77(11), 221A-226A (2005).
4. Bristol D.R., Probabilities and sample sizes for the two one-sided tests procedure, commun statist theory meth. 22(7), 1953-1961 (1993).

Table S1. Optical parameters of zinc oxide nanoflowers with different type of cells (ZnO-NFs-T98G, ZnO-NFs-SNU-80, ZnO-NFs- (ZnO-NFs-T98G, ZnO-NFs-SNU-80, ZnO-NFs-H460, ZnO-NFs-HEK, ZnO-NFs-MRC-5).

S.N.	Parameters	ZnO-NFs-T98G	ZnO-NFs-SNU-80	ZnO-NFs-H460	ZnO-NFs-HEK	ZnO-NFs-MRC-5
**1.**	Temperature of solutions	25 ± 1ºC	25 ± 1ºC	25 ± 1ºC	25 ± 1ºC	25 ± 1ºC
**2.**	Wavelength(nm)	420	420	420	420	420
**3.**	Spectra range (nm)	200-650	200-650	200-650	200-650	200-650
**4.**	Beer' s law limit (μg/mL)	0.5-2.1 μg/mL	0.5-2.1 μg/mL	0.5-2.1 μg/mL	0.5-2.2 μg/mL	0.5-2.2 μg/mL
**5.**	Molar absorptivity (ϵ) (L/mol/cm)	0.915×10^2	2.0×10^2	2.50×10^2	2.70×10^2	3.20×10^2
**6.**	Linear regression equation	A=-0.0177+ 0.0987C	A=0.0059 +0.1994C	A=-0.0122 +0.2504C	A=-0.0060 +0.2743C	A=-0.0224 +0.3238C
**7.**	$\pm ts_a$	5.102×10^{-4}	2.127×10^{-3}	4.046×10^{-3}	3.89×10^{-3}	4.65×10^{-3}
**8.**	$\pm ts_b$	3.503×10^{-4}	1.463×10^{-3}	2.784×10^{-3}	2.67×10^{-3}	3.20×10^{-3}
**9.**	Correlation coefficient (r^2)	0.994	0.994	0.992	0.995	0.996
**10.**	Variance (So^2) of calibration line	2.672×10^{-5}	1.138×10^{-4}	2.611×10^{-4}	2.013×10^{-4}	2.06×10^{-4}
**11.**	Detection limit (μg/mL)	0.172	0.176	0.213	0.170	0.146
**12.**	Quantitation limit (μg/mL)	0.523	0.535	0.645	0.517	0.443

Table S2. The intra-day and inter-day precision and accuracy data's of ZnO-NFs-T98G, ZnO-NFs-SNU-80, ZnO-NFs-H460, ZnO-NFs-HEK293, ZnO-NFs-MRC-5) obtained by the proposed method (n = 5)

S. N.	ZnO nanomaterials	Consecutive days	Added (µg/mL)	Found (µg/mL)	Standard analytical Error (SAE)[a]	Precision RSD[b] (%)	CL[c]
		Intraday	0.483	0.489	0.002	1.12	0.005
			1.496	1.498	0.006	1.19	0.016
**1.**	ZnO-NFs-T98G		2.033	2.030	0.005	0.60	0.013
		Interday	0.483	0.483	0.003	1.46	0.008
			1.496	1.490	0.007	0.94	0.019
			2.033	2.002	0.007	0.89	0.019
		Intraday	0.576	0.573	0.001	0.43	0.002
			1.534	1.530	0.003	0.46	0.008
**2.**	ZnO-NFs-SNU-80		2.035	2.041	0.003	0.32	0.008
		Interday	0.576	0.572	0.003	1.13	0.008
			1.534	1.522	0.004	0.55	0.011
			2.035	2.018	0.007	0.81	0.019
		Intraday	0.547	0.548	0.007	0.30	0.019
			1.462	1.466	0.002	0.37	0.005
**3.**	ZnO-NFs-H-460		2.045	2.052	0.005	0.63	0.013
		Interday	0.547	0.543	0.001	0.61	0.002
			1.462	1.448	0.005	0.90	0.013
			2.045	2.030	0.004	0.49	0.011
		Intraday	0.550	0.557	0.002	1.05	0.005
			1.589	1.594	0.007	0.75	0.019
**4.**	ZnO-NFs-HEK		2.063	2.078	0.006	0.71	0.016
		Interday	0.550	0.545	0.003	1.29	0.008
			1.589	1.576	0.005	0.72	0.013
			2.063	2.058	0.015	1.69	0.041
		Intraday	0.516	0.517	0.002	0.95	0.005
			1.474	1.476	0.004	0.60	0.011
**5.**	ZnO-NFs-MRC-5		2.045	2.052	0.005	0.63	0.013
		Interday	0.516	0.511	0.002	1.20	0.005
			1.474	1.456	0.007	1.15	0.019
			2.045	2.026	0.010	1.18	0.027

[a]SAE Standard analytical error.

[b]R.S.D. %, percentage relative standard deviation

[c]CL. Confidence limit at 95% confidence level (n=5)

Table S3. IC50 values (μg/mL) evaluated from MTT assay on H-460 (lung cancer), T98G (brain cancer), SNU-80 (thyroid cancer), HEK293 (non-malignant embryonic kidney) and MRC-5 (non-malignant lung fibroblast) cells treated with NPls, NSs, NRs and NFs for 24 h. Results are given as Mean (± SD), n=3.

Nano-structure	Cancerous cell lines			Non-cancerous Cell lines	
	**T98G**	**SNU-80**	**H-460**	**HEK**	**MRC-5**
ZnO-Pls	47.90 (±8.56)	74.0 (±6.21)	102.14 (±8.12)	86.62 (±13.83)	109.32 (±14.02)
ZnO-NSs	38.26 (±7.72)	47.88 (±15.45)	64.06 (±11.15)	112.19 (±13.81)	150.55 (± 12.15)
ZnO-NRs	36.80 (±2.57)	44.89 (±6.24)	94.94 (±9.22)	120.54 (±8.38)	157.20 (±15.17)
ZnO-NFs	48.62 (±5.32)	55.03 (±19.71)	107.97 (±11.48)	138.21 (±4.08)	185.15 (±8.40)

Table S4. List of RT^2 qPCR assays used for PCR analysis used in this study.

S.N.	Catalog No.	Product Description	Gene code
1.	PPH00073G	RT^2 qPCR Primer Assay for ACTB	Beta Actin (Human)
2.	PPH00107C	RT^2 qPCR Primer Assay for CASP3	CASP3 (Human)
3.	PPH00353B	RT^2 qPCR Primer Assay for CASP9	CASP9 (Human)
4.	PPH00686B	RT^2 qPCR Primer Assay for PARP1	PARP1 (Human)
7.	PPH20675F	RT^2 qPCR Primer Assay for CYCS	CYCS (Human)
8.	PPH00213F	RT^2 qPCR Primer Assay for TP53	p53 (Human)
